# Geometry and bone mineral density determinants of femoral neck strength changes following exercise

**DOI:** 10.1007/s10237-022-01642-w

**Published:** 2022-10-21

**Authors:** Dermot O’Rourke, Belinda R. Beck, Amy T. Harding, Steven L. Watson, Peter Pivonka, Saulo Martelli

**Affiliations:** 1 Medical Device Research Institute, College of Science and Engineering, Flinders University, Adelaide, Australia; 2 School of Mechanical, Medical and Process Engineering, Queensland University of Technology, Brisbane, Australia; 3 Menzies Health Institute Queensland, Gold Coast, Australia; 4 School of Health Sciences and Social Work, Griffith University, Gold Coast, Australia; 5 The Bone Clinic, Brisbane, Australia; 6 Gold Coast University Hospital, Gold Coast, Australia

**Keywords:** Exercise, Osteoporosis, DXA, Bone adaptation, Fracture prevention

## Abstract

Physical exercise induces spatially heterogeneous adaptation in bone. However, it remains unclear where the changes in BMD and geometry have the greatest impact on femoral neck strength. The aim of this study was to determine the principal BMD-and-geometry changes induced by exercise that have the greatest effect on femoral neck strength. Pre- and post-exercise 3D-DXA images of the proximal femur were collected of male participants from the LIFTMOR-M exercise intervention trial. Meshes with element-by-element correspondence were generated by morphing a template mesh to each bone to calculate changes in BMD and geometry. Finite element (FE) models predicted femoral neck strength changes under single-leg stance and sideways fall load. Partial least squares regression (PLSR) models were developed with BMD-only, geometry-only, and BMD-and-geometry changes to determine the principal modes that explained the greatest variation in neck strength changes. The PLSR models explained over 90% of the strength variation with 3 PLS components using BMD-only (*R*^2^ > 0.92, RMSE < 0.06 N) and 8 PLS components with geometry-only (*R*^2^ > 0.93, RMSE < 0.06 N). Changes in the superior neck and distal cortex were most important during single-leg stance while the superior neck, medial head, and lateral trochanter were most important during a sideways fall. Local changes in femoral neck and head geometry could differentiate the exercise groups from the control group. Exercise interventions may target BMD changes in the superior neck, inferior neck, and greater trochanter for improved femoral neck strength in single-leg stance and sideways fall.

## Introduction

Osteoporotic hip fractures are a considerable cause of morbidity and mortality (Abrahamsen et al. 2009) and occur in specific local regions (de Bakker et al. 2009) where strain exceeds tissue strength. Focal bone loss in osteoporosis has been found to play a key role in determining fracture risk and fracture location (Poole et al. 2017). Meanwhile, the mechanical loading associated with exercise can cause higher than normal strain in certain locations which elicits a spatially heterogeneous adaptative response in the bone to improve resistance to future strain (Lang et al. 2014). However, the relative contributions of spatially heterogeneous adaptations in BMD and geometry on femoral neck strength are not well understood.

There are contrasting opinions on where spatially heterogeneous adaptation in the proximal femur has the greatest impact on its strength. Increased hip fragility has been associated with thinning of cortical bone in the superior femoral neck (Martelli et al. 2021) and studies have identified exercises that induced high focal strains (Martelli et al. 2020) and increased thickness in this region (Allison et al. 2015). However, others have found adaptation localised to within the inferior neck regions increased strength in single-leg stance with a 4.1% dominant to non-dominant leg difference found for athletes who exhibited greater vBMD on the medial side in their dominant leg (Warden et al. 2020) and a 60% greater cortical thickness in the inferior region of the femoral neck in athletes performing sports with high-impact loading compared to a non-athletic control group (Nikander et al. 2009). Therefore, adaptation in the inferior neck has been suggested to increase strength as a result of an inferior shift of the neutral axis providing indirect protection of the superior neck (Warden et al. 2020). Meanwhile, it has been suggested geometry plays a role in determining strength with the combination of BMD and measures of femur geometry on planar radiographic images having been shown to improve the assessment of hip fracture risk and fracture type compared to BMD alone (Kaptoge et al. 2008; Pulkkinen et al. 2004). Yet, it remains unclear how changes in geometry and BMD induced by exercise determine femoral neck strength.

Advances in technology have enabled assessment of 3D proximal femur geometry and volumetric BMD (vBMD) distribution from planar DXA images (Humbert et al. 2017). Previously, we developed a method for assessing changes in vBMD and femoral neck strength from 3D-DXA images in large cohorts and demonstrated the method can assess the effect of an exercise intervention (O'Rourke et al. 2021). The methodology established correspondence between bones so that changes in spatial BMD and geometry could be assessed and bone strength could be estimated using finite element (FE) models derived from the volume images. FE estimations of bone strength based on 3D-DXA images have been validated against experimental results (Grassi et al. 2017) and shown to improve discrimination of fracture and non-fracture cases (Wills et al. 2019). Therefore, the established method can provide information of variation in BMD and geometry over the hip volume while the analysis of the images via FE modelling can provide information of femoral neck strength (Schileo et al. 2014; Taylor et al. 2017). Partial least squares regression (PLSR) is a statistical method that constructs new predictor variables, known as PLS components, as linear combinations of principal modes of variation maximising the fraction of the variance of a response variable explained by a multiple regression model (Wold et al. 2001). Therefore, PLS may enable quantification of the contribution of spatially heterogeneous BMD-and-geometry variations to femoral neck strength.

The primary aim of the current study was to determine the features of geometry and spatial BMD changes that had the greatest impact on femoral neck strength changes induced by exercise. A secondary aim was to compare BMD-and-geometry changes caused by an established exercise protocol to controls based on those BMD-and-geometry changes most associated with changes in strength. To this purpose, we analysed pre- and post-intervention 3D-DXA images pooled from male participants in an 8-month semi-randomised controlled trial of exercise programmes to reduce fragility fracture risk (Harding et al. 2020). The current study developed PLSR models to determine the principal modes of geometry and spatial BMD changes that explained the greatest variance in femoral neck strength changes during a single-leg stance and sideways fall. The principal modes of geometry-and-BMD changes were then compared between exercise and control groups.

## Methods

### 3D-DXA

Pre- and post-exercise intervention DXA scans (Medix DR, Medilink, France) were obtained of the non-dominant proximal femur from male participants with osteoporosis and osteopenia (67 ± 7 years) in the LIFTMOR-M 8-month semi-randomised controlled exercise intervention trial (Harding et al. 2020). The trial was approved by the Griffith University Human Research Ethics Committee (AHS/07/14/HREC) and all participants provided written informed consent. The participants were from three groups: a high-intensity progressive resistance and impact training group (HiRIT, *n* = 34), a machine-based isometric axial compression group (IAC, *n* = 33), and a control group of sex- and age-matched participants from the same community (67 ± 6 years) (*n* = 25). The HiRIT programme consisted of multi-joint, compound movement, high-intensity progressive resistance training, and high-impact jumping exercises. The IAC programme incorporated self-initiated near-maximal 5-s isometric contractions performed for the chest press, leg press, core pull, and vertical lift exercises using the bioDensity™ system. The trial protocol has been published describing the exercise interventions and control group activities (Harding et al. 2017).

3D-DXA images (1 × 1 × 1 mm) calibrated to equivalent BMC levels were obtained from the DXA scans with 3D-SHAPER software (v.2, Galgo Medical, Barcelona, Spain). The software delivers a 3D volume of images of the proximal femur from a planar DXA image using a 3D statistical shape and appearance model (SSAM) of the proximal femur built from a database of 111 Caucasian individuals (56 ± 12 years) with no signs of skeletal disease other than osteoporosis. Femur models generated from the 3D-DXA images produced by the software have shown a 0.93 mm mean point-surface distance to corresponding femurs generated from quantitative computed tomography (QCT) and a correlation coefficient = 0.95 for vBMD measurements (Humbert et al. 2017).

### BMD-and-geometry changes

Mesh morphing was performed on each proximal femur geometry using iterative closest point algorithms to establish all meshes with node numbering and connectivity correspondence to calculate pre- and post-exercise changes. The proximal femur surface geometry was retrieved from each 3D-DXA image through threshold-based segmentation and meshed with triangular elements. The triangulated meshes underwent 5 iterations of smoothing using curvature flow smoothing (Desbrun et al. 1999) (MATLAB 2018b, The MathWorks Inc., MA, USA). A separate template surface mesh of the proximal femur geometry was characterised by a triangular surface mesh (33,954 elements) where 90% of the elements had a Jacobian greater than 0.8 and edge sizes of 1.25–1.75 mm. A template volume mesh of linear tetrahedral elements (205,233 elements) was generated from the surface using an advancing front algorithm (Hypermesh 14.0, Altair Engineering Inc., Troy, USA).

Iterative closest point (ICP) based registration was used to morph the template surface mesh over the geometry of each bone. First, the template surface mesh was rigidly registered to the target bone surface by aligning the principal axes of inertia of the template and the target surface. The registration was then optimised with a rigid ICP algorithm so that rotation, translation, and scaling of the template vertices were iteratively calculated as solutions to a weighted least squares minimisation problem by Singular Value Decomposition (SVD). The algorithm converged when the root mean squared error (RMSE) between the previous and current template vertices dropped below a threshold value (< 10^−5^ mm). Secondly, non-rigid registration modelled as a sum of Gaussian Radial Basis Functions (G-RBF) with 10 iterations elastically deformed the template surface mesh to match the geometry of the target surface mesh. Following registration of the template surface mesh, a custom FE solution was then implemented in which the displacements at the nodes on the surface of the template volume mesh were imposed to equal those calculated while registering the surface meshes (MATLAB 2020b, The MathWorks Inc., MA, USA). This algorithm delivered morphed volume meshes with node numbering and connectivity correspondence.

The bone density was mapped to the morphed template volume mesh from the 3D-DXA image. To achieve this, the bone density distribution in the image was integrated over each element volume using Gaussian quadrature to deliver the density distribution in the mesh. Changes in geometry were expressed as the pre- and post-exercise changes in x, y, and z coordinates (mm) of corresponding nodes in the morphed volume meshes to represent each bone instance in the dataset. BMD changes were expressed as the change in BMD (g/cm^3^) at corresponding elements between the pre- and post-exercise volume meshes.

### Femoral neck strength changes

FE models were built from the morphed volume mesh of each bone to predict the femoral neck strength under load. Bone was assumed to be linear-elastic and have locally isotropic properties. An empirical relationship was used to convert the density (g/cm^3^) in the images to elastic modulus (MPa) (Morgan et al. 2003) with a correction for bone ash density (Schileo et al. 2008a):

1 $$E = {14644 } \times \rho^{{{1}.{49}}}.$$

The Poisson’s ratio was set to 0.3.

A local femoral coordinate system was defined on the proximal femur by first defining two rings of nodes on the shaft above and below the lesser trochanter. The line joining the centre points of the node rings defined the Z-axis. A Z-X plane was defined by establishing a temporary axis between the proximal femur centroid above the lesser trochanter and the centre of the femoral head. The Y-axis was defined as the cross-product of the Z-axis and temporary axis in the Z-X plane. The X-axis was defined as the cross-product of the Z-axis and Y-axis (O'Rourke et al. 2021).

**Fig. 1:**
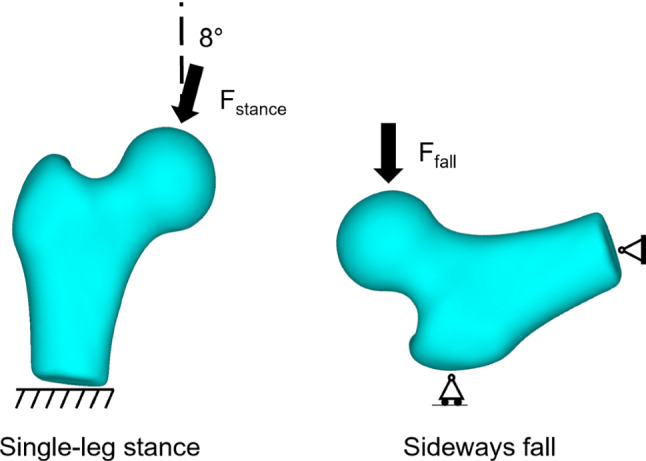
Single-leg stance and sideways fall loading conditions simulated to calculate the change in femoral neck strength

Two sets of loading configurations were used: a static single-leg stance and a sideways fall. Single-leg stance was simulated by constraining the distal end of the femur. A nominal load of 100 N lying in the frontal plane and passing through the femoral head centre at 8° abduction from the local vertical (Z) axis was applied to simulate a static single-leg stance (Cristofolini et al. 2007). The force was distributed over a 5 mm diameter node patch on the superior femoral head surface to minimise numerical artefacts. The sideways fall loading was simulated with a nominal load of 100 N distributed over a 5 mm diameter node patch on the medial side of the femoral head directed laterally along the local medial–lateral (X) axis. The distal extremity of the femur was free to rotate about the anterior–posterior axis and the surface of the greater trochanter was constrained in the medial–lateral direction (Grassi et al. 2012) (Fig. 1). All simulations were performed in ANSYS (Version 19.1, ANSYS Inc., PA, USA).

Femoral neck strength was calculated for each loading configuration using a principal strain criterion (Schileo et al. 2008b). The maximum and minimum principal bone strains were recorded for each element on the external surface of the femoral neck (Supplementary Information) and averaged over a 3 mm spherical radius to avoid local artefact (Schileo et al. 2014). The risk of fracture (RF) was calculated as the ratio between the principal tensile and compressive strain (*ε*_MAX_) and the asymmetric elastic limit values of bone in compression (*ε*_LIM_ = 10,000 µε) and tension (*ε*_LIM_ = 7000 µε) (Bayraktar et al. 2004). Strength (Fs) was determined as the nominal load applied (*F*_nominal_) (100 N) over the maximum RF (*RF*_max_):

2 $$F_{s} = F_{{{\text{nominal}}}} /RF_{{{\text{max}}}}.$$

Changes in single-leg stance and sideways fall femoral neck strength pre- and post-exercise intervention were expressed as a percentage.

### Data analysis

Partial least squares regression (PLSR) was applied on the changes in proximal femur geometry, BMD distribution, and femoral neck strength with all participants. The analysis delivers a series of BMD-and-geometry change maps ranked according to the amount of variation in strength changes explained. PLSR models were developed with: (1) combined geometry and BMD, (2) geometry-only, and (3) BMD-only. All variables were standardised before applying the PLSR by calculating their z-score. The PLSR was solved with the SIMPLS algorithm (MATLAB 2020b, The MathWorks Inc., MA, USA). The compactness of the PLSR model was determined by how many PLS components were required to explain over 90% of the variation in strength changes where fewer components indicated a stronger relationship to strength. The error in the PLSR model was determined with the coefficient of determination (*R*^2^) and root mean square error (RMSE) using tenfold cross-validation.

The relative importance of the geometry-and-BMD variable to the PLSR model was assessed using the Variable Importance in Projection (VIP) scores, which estimate the relative contribution of the BMD or geometry change at each point in the femur used in the PLSR model. The VIP scores are calculated as a weighted sum of the squared correlations between the PLS components (Wold et al. 1993). A VIP score > 1.5 was used as a threshold for important variable selection in the BMD-only and geometry-only models.

The effect of exercise on femoral neck strength was assessed by comparing changes in vBMD in the control group to corresponding changes in the HiRIT and the IAC group using a one-way ANOVA. The same test was then repeated for the per cent changes in predicted femoral neck strength and PLS component scores. Where the F value for a given parameter was found to be statistically significant (*p* < 0.05), it was followed by unpaired t-tests with a Bonferroni adjustment for multiple comparisons.

## Results

**Fig. 2:**
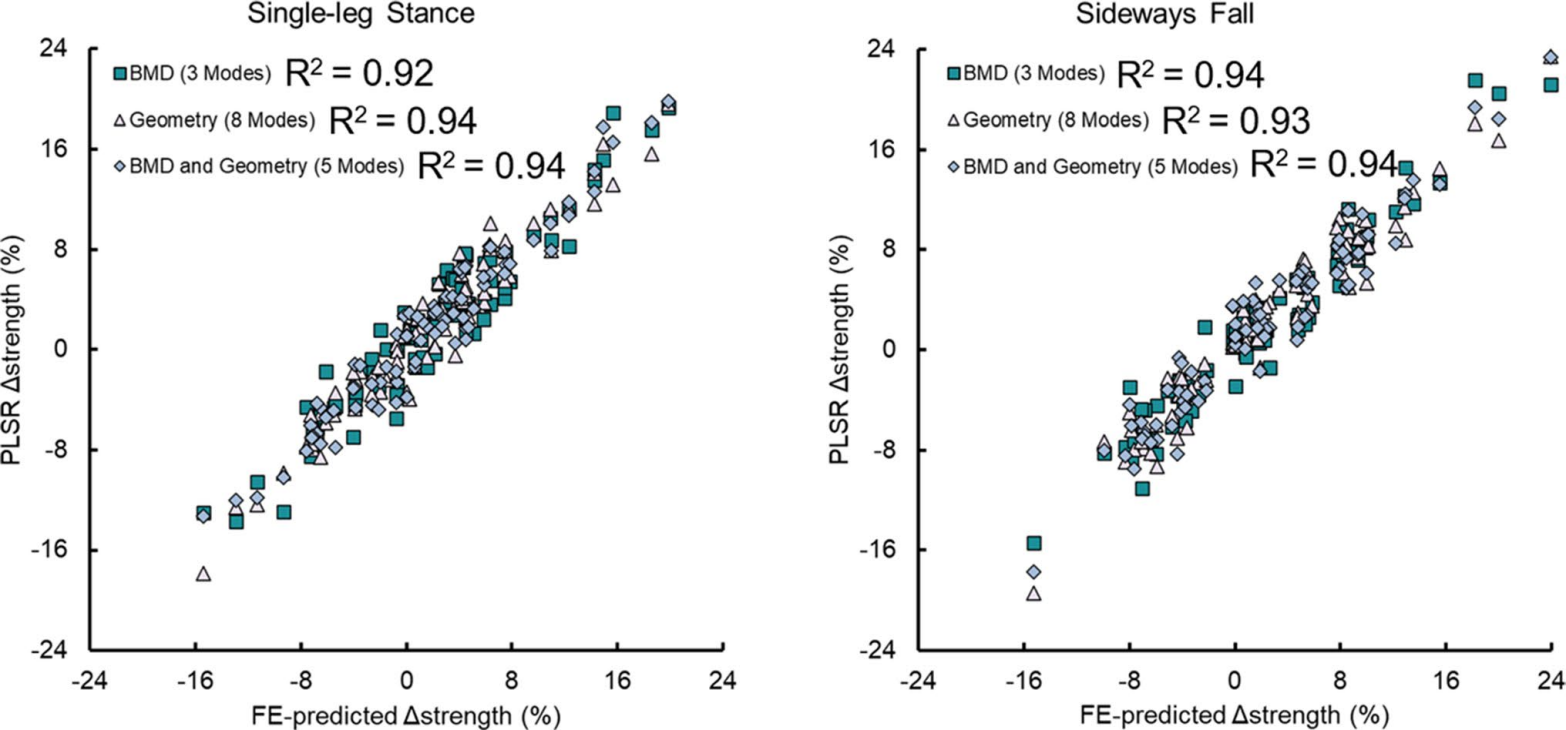
Regression plots of the BMD-only, geometry-only, and BMD-and-geometry partial least squares regression (PLSR) models for changes in finite element-predicted femoral neck strength for single-leg stance and sideways fall loading conditions in all participants (*n* = 92). The PLSR models in the plots were built with the specified number of modes which explained over 90% of the variation in strength

**Fig. 3:**
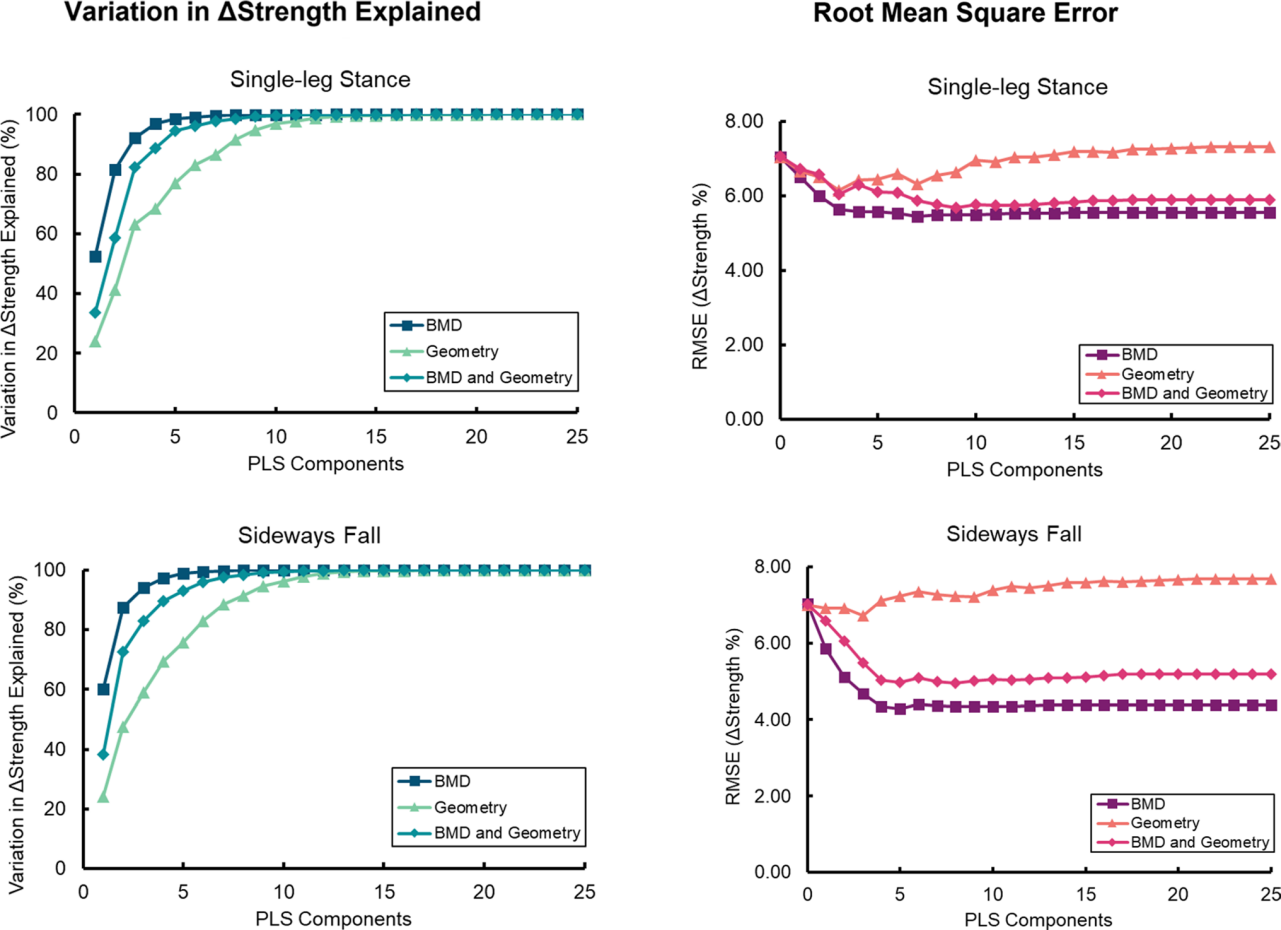
Cumulative variation explained of the strength changes in single-leg stance and sideways fall strength for the partial least squares (PLS) components in the partial least squares regression (PLSR) models (*n* = 92). Root mean square error (RMSE) of the PLSR models for predicting strength changes with an increasing number of PLS components for both loading conditions. RMSE was determined at each iteration using tenfold cross-validation

The BMD-only PLSR model explained over 90% of the strength variation in single-leg stance and sideways fall across all participants with 3 PLS components (*R*^2^ > 0.92, RMSE < 0.06 N) and over 95% of the variation with 4 PLS components (*R*^2^ = 0.97, RMSE < 0.06 N) (Fig. 2). The first PLS component explained the greatest proportion of the variation in strength accounting for 53% and 60% of the strength variation in the single-leg stance and sideways fall, respectively. Meanwhile, the geometry-only models explained over 90% of the strength variation in single-leg stance and sideways fall with 8 PLS components (*R*^2^ > 0.93, RMSE < 0.06 N). The first PLS component accounted for 24% of the variability in each loading condition. The combined BMD-and-geometry models were closer to the BMD-only models, explaining 90% of the bone strength variation in both single-leg stance and sideways fall with 5 PLS components each (*R*^2^ > 0.93, RMSE < 0.06 N). The first PLS component explained a level of variation in strength closer to the geometry-only model, with 43% variation explained in single-leg stance strength and 38% in sideways fall, whereas over 90% of the total variation was explained with 5 modes, which was more like the BMD-only model (Fig. 3).

**Fig. 4:**
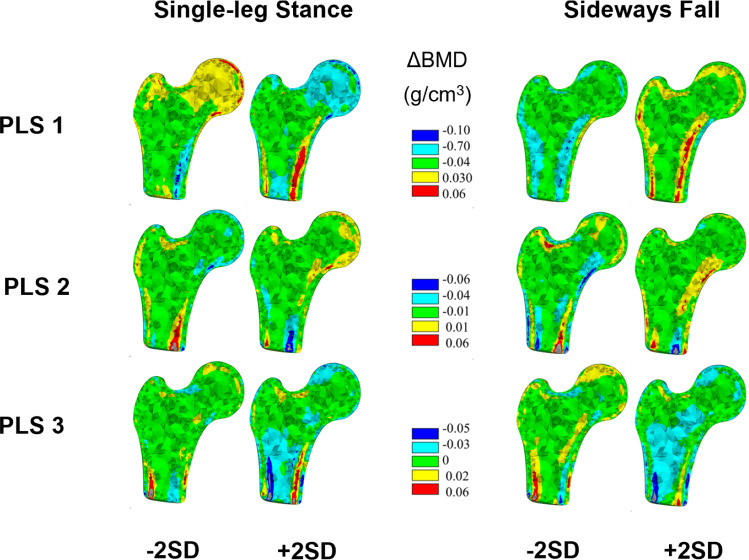
Changes in BMD maps for the first three partial least squares (PLS) components in the BMD-only partial least squares regression (PLSR) model. The BMD changes captured are shown for each PLS component perturbed by ± 2 standard deviations (SD) from the mean

**Fig. 5:**
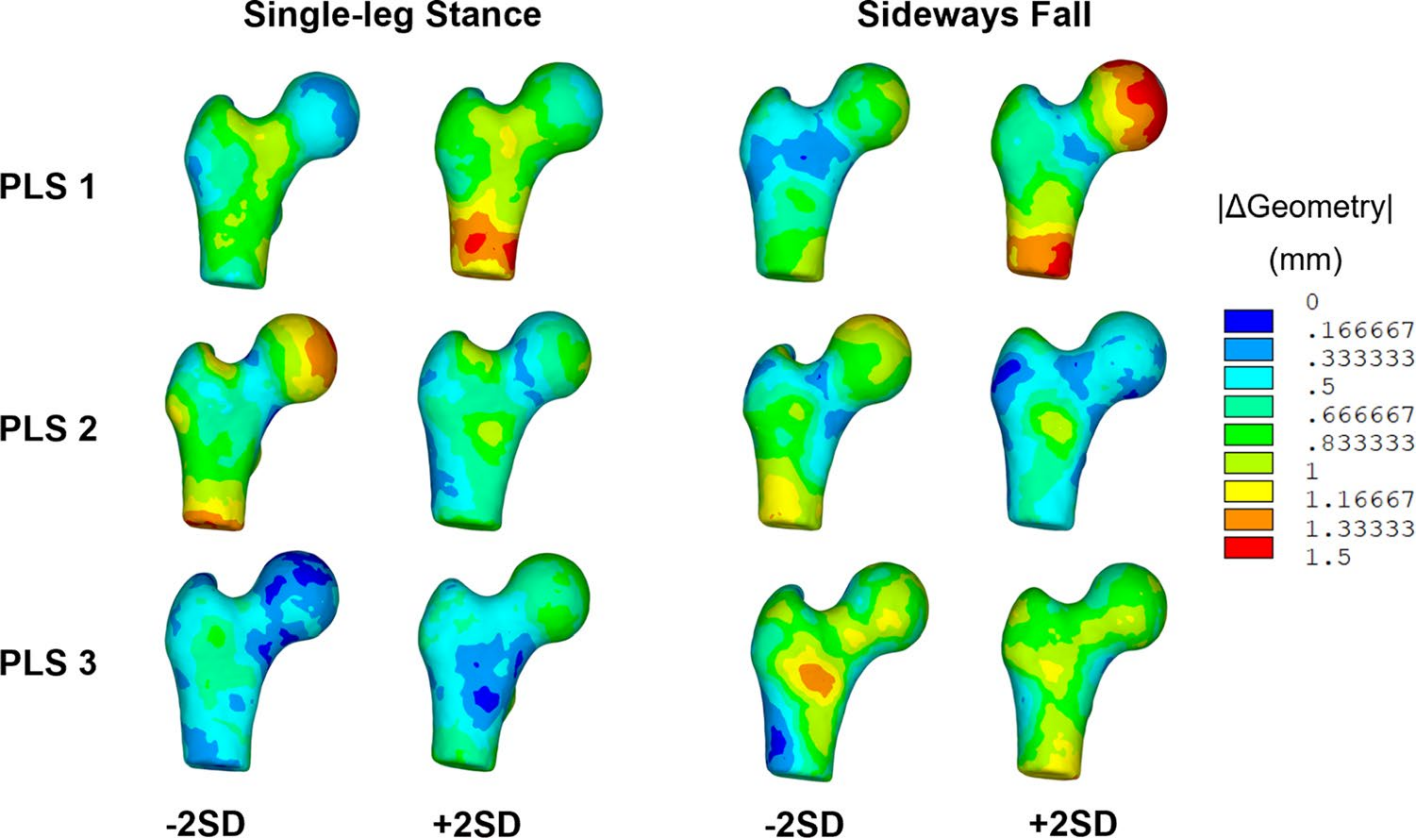
Changes in geometry maps for the first three partial least squares (PLS) components in the geometry-only partial least squares regression (PLSR) model. The geometry values represent the magnitude of the changes in x, y, and z coordinates for each node when the PLS components are perturbed ± 2 standard deviations (SD) from the mean

**Fig. 6:**
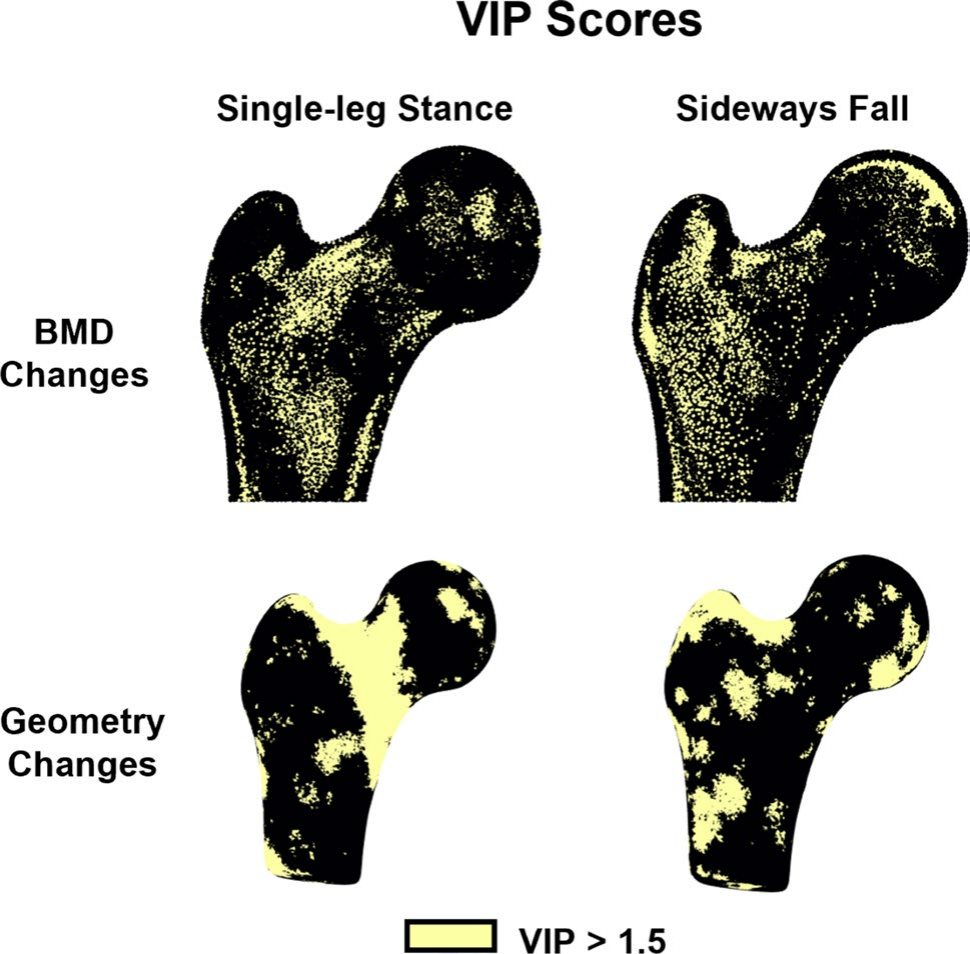
Variable importance in projection (VIP) scores for the BMD-only and geometry-only partial least squares regression (PLSR) models indicating the key regions in the proximal femur that contributed to the PLSR models

The first three modes of variation of the BMD-only models displayed similar patterns of BMD changes for both single-leg stance and sideways fall. The largest BMD changes (± 0.1 g/cm^3^) were found in the cortex distal to the femoral neck for the first mode of variation. The second mode displayed focal changes (± 0.06 g/cm^3^) in the distal cortex, superior and inferior neck cortex, and the medial femoral head. The third mode showed the largest BMD changes (± 0.05 g/cm^3^) in the distal cortex and superior femoral head, while BMD changes in the superior neck were slightly larger for the sideways fall load case as compared to the single-leg stance load case (Fig. 4). Similarly, the first two modes of variation of the geometry-only models for single-leg stance and sideways fall displayed the largest changes in the proximal femoral shaft and femoral head and the femoral neck (Fig. 5). However, the VIP scores indicated that the most important BMD changes for femoral neck strength differed between sideways fall and single-leg stance. During sideways fall, the most important regions were those of the superior femoral head and lateral trochanteric region while during single-leg stance, the most important BMD changes were those of the superior neck and the distal cortex. The VIP scores for geometry changes confirmed the importance of the femoral neck during single-leg stance and the trochanteric and head regions during a sideways fall (Fig. 6).

Comparing vBMD and strength changes in the HiRIT group, the IAC group, and the controls, there were no statistically significant differences between groups for vBMD changes (*F* = 1.05, *p* = 0.36) nor in strength changes either for single-leg stance (*F* = 0.26, *p* = 0.77) and sideways fall (*F* = 0.83, *p* = 0.77) as determined by one-way ANOVA (Table 1). However, differences were observed between the control group and both the HiRIT and IAC groups using the PLS modes in the geometry-only, and the geometry-and-BMD models. Specifically, a Bonferroni post hoc test indicated that both the HiRIT and IAC groups had significantly higher PLS component 2 scores in single-leg stance and PLS component 1 scores in sideways fall (Supplementary Information). No difference in PLS component scores was found between the HiRIT and IAC groups.

**Table 1:** Mean ± standard deviation for total hip volumetric BMD, single-leg stance strength (SLS), and sideways fall strength (SF) changes in the high-intensity progressive resistance and impact (HiRIT) group, the isometric axial compression (IAC) exercise group, and control group

	Control (*n* = 25)			HiRIT (*n* = 34)			IAC (*n* = 33)		
	Baseline	Follow-up	% change	Baseline	Follow-up	% change	Baseline	Follow-up	% change
vBMD (g/cm^3^)	0.29 ± 0.04	0.29 ± 0.04	− 0.1 ± 2.4	0.27 ± 0.03	0.28 ± 0.04	0.93 ± 2.6	0.272 ± 0.025	0.274 ± 0.025	0.87 ± 3.3
SLS (N)	4021 ± 499	4093 ± 520	1.9 ± 6.2	3801 ± 632	3837 ± 651	1.2 ± 8.6	3770 ± 574	3850 ± 531	2.5 ± 5.8
SF (N)	1699 ± 340	1704 ± 332	0.6 ± 6.2	1501 ± 291	1533 ± 301	2.3 ± 7.2	1523 ± 266	1564 ± 273	2.9 ± 7.4

## Discussion

The aim of the current study was to determine the principal modes of variation of BMD-and-geometry changes with the greatest effect on femoral neck strength in participants who underwent an established exercise treatment. We found similar variations of BMD changes in the cortical, superior neck, and femoral head explained most of the variation in strength for single-leg stance and sideways fall. Nevertheless, the relative importance of different regional changes was loading-specific, with the superior neck and distal cortex being most important during single-leg stance while the superior neck, medial head, and lateral trochanter were most important during sideways fall. The present results can inform the design of exercise programmes on the principal target locations to enhance the strength of the proximal femur during single-leg stance and sideways fall loading.

The principal modes of BMD-and-geometry variation explained, either separately or conjointly, more than 90% of the variation in femoral neck strength across the participants and their response to exercise. These results thereby indicated the interdependence between BMD and geometry in determining strength. Yet, the BMD-only model was more compact than both the geometry-only and the BMD-and-geometry models supporting the notion of a dominant effect of BMD on proximal femur strength over that of geometry (Cheng et al. 1997; Roberts et al. 2010). Interestingly, the spatially heterogeneous BMD changes represented by the PLS modes of variation were able to identify differences between the HiRIT, the IAC group, and the controls attributable to the exercise programme as opposed to the non-significant changes found in corresponding measurements of volumetric BMD and predictions of strength. The first two modes of BMD-and-geometry variation displayed similar cortical, superior neck, and femoral head changes for both single-leg stance and sideways fall loading. However, the most important regions, as indicated by the VIP scores, associated with strength shifted from the neck and distal cortex during single-leg stance to the medial head and lateral trochanter during a sideways fall consistent with the respective shift of the load from longitudinal to transversal compression. Therefore, the PLS modes provided a more sensitive measurement than vBMD and strength measurements for the assessment of bone changes following exercise. They also provided insights into understanding the different bone compartments response to exercise, their interactions in supporting the femur during loading, and how the response of the compartments differ due to loading direction. These findings may help advance fragility fracture prevention and prediction methods.

The current study found that BMD changes had a greater contribution to the changes in strength with over 90% of the variation explained with 3 PLS components in accordance with previous investigations indicating low BMD as the strongest risk factor for fracture (Roberts et al. 2010). The geometry-only models could explain over 90% of the variation in strength over with 8 PLS components but combining both into the geometry-and-BMD model did not improve the prediction of strength over BMD alone as has been observed previously (Kaptoge et al. 2008; Pulkkinen et al. 2006). The thin superior femoral neck cortex was consistently one of the most important regions for predicting proximal femur strength during both single-leg stance and sideways fall in agreement with previous findings (Mayhew et al. 2005) that the superior neck is the weakest link of the proximal femur and a common fracture location in both clinical (LaCroix et al. 2010) and laboratory settings (Palanca et al. 2021). The loading-specific VIP maps provided here are consistent with the exercise-induced bone accrual within the superior femoral head, inferior neck, medial intertrochanteric, and greater trochanter causing a proximal femur strength increase varying from 4.9% in sideways fall to 19% in single-leg stance (Fuchs et al. 2021), supporting the use of exercise prescription that promotes a more uniform response of proximal femur strength to exercise (Abe et al. 2016) (Martelli et al. 2020). Furthermore, the BMD changes in the inferior neck impacting strength in single-leg stance are consistent with earlier observations in baseball pitchers, which were considered to indirectly protect the weakest link in the superior neck by causing an inferior shift of the neutral axis (Warden et al. 2020). The VIP scores in the present study also indicate that the inferior neck is an important region during single-leg stance but much less so during sideways fall.

The strength of our study lies in the comprehensive analysis technique SSAM with FE simulations to estimate femoral neck strength in combination with use of PLSR models. However, there are limitations that warrant acknowledgement. Firstly, the 3D-DXA technology used in this study provided volumetric BMD distribution in each participant reconstructed from planar DXA images at two time points thereby enabling the present study into the relationship between geometry, BMD, and strength in the participants. The relatively low repeatability of the technology should be taken into consideration when applied on a participant-by-participant base (O'Rourke et al. 2021). Secondly, the study focused on bone strength while exercise prescription should be based on broader health considerations (Beck et al. 2017). As such, exercise prescription should consider the equivalence of the HiRIT and IAC exercise programmes for promoting femoral neck strength reported here with caution and in conjunction with broader considerations of general health of participants (Beck et al. 2017). Finally, this study only considered middle-aged and older men and does not consider sex-related differences. Women are at greater risk of hip fragility fracture and may not show the same patterns of response to exercise as men, meaning our results may not be generalisable to the opposite sex (Fuchs et al. 2021).

In conclusion, the current study determined the principal BMD-and-geometry changes induced by exercise that explained most of the variation in femoral neck strength. Changes to BMD in the superior neck, inferior neck, and greater trochanter primarily explained the variation in neck strength changes seen in the group of middle-aged and older men with osteopenia and osteoporosis in single-leg stance and sideways fall loading. Local changes in femoral neck and head geometry could differentiate the exercise groups from the control group, but not in predicted strength changes. Exercise interventions may target BMD changes in the superior neck, inferior neck, and greater trochanter for improved femoral neck strength in single-leg stance and sideways fall.

## References

[CR1] Abe S, Narra N, Nikander R, Hyttinen J, Kouhia R, Sievänen H (2016). Exercise loading history and femoral neck strength in a sideways fall: A three-dimensional finite element modeling study. Bone.

[CR2] Abrahamsen B, Van Staa T, Ariely R, Olson M, Cooper C (2009). Excess mortality following hip fracture: a systematic epidemiological review. Osteoporos Int.

[CR3] Allison SJ (2015). The influence of high-impact exercise on cortical and trabecular bone mineral content and 3D distribution across the proximal femur in older men: a randomized controlled unilateral intervention. J Bone Mineral Res.

[CR4] Bayraktar HH, Morgan EF, Niebur GL, Morris GE, Wong EK, Keaveny TM (2004). Comparison of the elastic and yield properties of human femoral trabecular and cortical bone tissue. J Biomech.

[CR5] Beck BR, Daly RM, Singh MAF, Taaffe DR (2017). Exercise and Sports Science Australia (ESSA) position statement on exercise prescription for the prevention and management of osteoporosis. J Sci Med Sport.

[CR6] Cheng XG, Lowet G, Boonen S, Nicholson PHF, Brys P, Nijs J, Dequeker J (1997). Assessment of the strength of proximal femur in vitro: relationship to femoral bone mineral density and femoral geometry. Bone.

[CR7] Cristofolini L, Juszczyk M, Martelli S, Taddei F, Viceconti M (2007). In vitro replication of spontaneous fractures of the proximal human femur. J Biomech.

[CR8] de Bakker PM, Manske SL, Ebacher V, Oxland TR, Cripton PA, Guy P (2009). During sideways falls proximal femur fractures initiate in the superolateral cortex: evidence from high-speed video of simulated fractures. J Biomech.

[CR9] Desbrun M, Meyer M, Schröder P, Barr AH (1999) Implicit fairing of irregular meshes using diffusion and curvature flow. In: Proceedings of the 26th annual conference on computer graphics and interactive techniques, SIGGRAPH 1999. pp 317–324. 10.1145/311535.311576

[CR10] Fuchs RK, Carballido-Gamio J, Keyak JH, Kersh ME, Warden SJ (2021). Physical activity induced adaptation can increase proximal femur strength under loading from a fall onto the greater trochanter. Bone.

[CR11] Grassi L, Schileo E, Taddei F, Zani L, Juszczyk M, Cristofolini L, Viceconti M (2012). Accuracy of finite element predictions in sideways load configurations for the proximal human femur. J Biomech.

[CR12] Grassi L, Väänänen SP, Ristinmaa M, Jurvelin JS, Isaksson H (2017). Prediction of femoral strength using 3D finite element models reconstructed from DXA images: validation against experiments. Biomech Model Mechanobiol.

[CR13] Harding AT, Weeks BK, Lambert C, Watson SL, Weis LJ, Beck BR (2020). A comparison of bone-targeted exercise strategies to reduce fracture risk in middle-aged and older men with osteopenia and osteoporosis: LIFTMOR-M semi-randomized controlled trial. J Bone Mineral Res.

[CR14] Harding AT, Weeks BK, Watson SL, Beck BR (2017). The LIFTMOR-M (Lifting Intervention for Training Muscle and Osteoporosis Rehabilitation for Men) trial: Protocol for a semirandomised controlled trial of supervised targeted exercise to reduce risk of osteoporotic fracture in older men with low bone mass. BMJ Open.

[CR15] Humbert L (2017). 3D-DXA: assessing the femoral shape, the trabecular macrostructure and the cortex in 3D from DXA images. IEEE Trans Med Imaging.

[CR16] Kaptoge S, Beck TJ, Reeve J, Stone KL, Hillier TA, Cauley JA, Cummings SR (2008). Prediction of incident hip fracture risk by femur geometry variables measured by hip structural analysis in the study of osteoporotic fractures. J Bone Miner Res.

[CR17] LaCroix AZ (2010). Hip structural geometry and incidence of hip fracture in postmenopausal women: what does it add to conventional bone mineral density?. Osteoporos Int.

[CR18] Lang TF (2014). Spatial heterogeneity in the response of the proximal femur to two lower-body resistance exercise regimens. J Bone Miner Res.

[CR19] Martelli S, Beck B, Saxby D, Lloyd D, Pivonka P, Taylor M (2020). Modelling human locomotion to inform exercise prescription for osteoporosis. Curr Osteoporos Rep.

[CR20] Martelli S, Giorgi M, Dall' Ara E, Perilli E (2021). Damage tolerance and toughness of elderly human femora. Acta Biomater.

[CR21] Mayhew PM (2005). Relation between age, femoral neck cortical stability, and hip fracture risk. Lancet.

[CR22] Morgan EF, Bayraktar HH, Keaveny TM (2003). Trabecular bone modulus-density relationships depend on anatomic site. J Biomech.

[CR23] Nikander R (2009). Targeted exercises against hip fragility. Osteoporo Int.

[CR24] O'Rourke D, Beck BR, Harding AT, Watson SL, Pivonka P, Martelli S (2021). Assessment of femoral neck strength and bone mineral density changes following exercise using 3D-DXA images. J Biomech.

[CR25] Palanca M, Perilli E, Martelli S (2021). Body anthropometry and bone strength conjointly determine the risk of hip fracture in a sideways fall. Ann Biomed Eng.

[CR26] Poole K et al (2017) Focal osteoporosis defects play a key role in hip fracture, vol 94. Elsevier Inc. 10.1016/j.bone.2016.10.020 10.1016/j.bone.2016.10.020 PMC5135225 27777119

[CR27] Pulkkinen P, Eckstein F, Lochmüller E-M, Kuhn V, Jämsä T (2006). Association of geometric factors and failure load level with the distribution of cervical vs. trochanteric hip fractures. J Bone Mineral Res.

[CR28] Pulkkinen P, Partanen J, Jalovaara P, Jämsä T (2004). Combination of bone mineral density and upper femur geometry improves the prediction of hip fracture. Osteoporos Int.

[CR29] Roberts BJ, Thrall E, Muller JA, Bouxsein ML (2010). Comparison of hip fracture risk prediction by femoral aBMD to experimentally measured factor of risk. Bone.

[CR30] Schileo E, Balistreri L, Grassi L, Cristofolini L, Taddei F (2014). To what extent can linear finite element models of human femora predict failure under stance and fall loading configurations?. J Biomech.

[CR31] Schileo E, Dall’Ara E, Taddei F, Malandrino A, Schotkamp T, Baleani M, Viceconti M (2008). An accurate estimation of bone density improves the accuracy of subject-specific finite element models. J Biomech.

[CR32] Schileo E, Taddei F, Cristofolini L, Viceconti M (2008). Subject-specific finite element models implementing a maximum principal strain criterion are able to estimate failure risk and fracture location on human femurs tested in vitro. J Biomech.

[CR33] Taylor M, Perilli E, Martelli S (2017). Development of a surrogate model based on patient weight, bone mass and geometry to predict femoral neck strains and fracture loads. J Biomech.

[CR34] Warden SJ (2020). Heterogeneous spatial and strength adaptation of the proximal femur to physical activity: a within-subject controlled cross-sectional study. J Bone Miner Res.

[CR35] Wills CR (2019). 3D patient-specific finite element models of the proximal femur based on DXA towards the classification of fracture and non-fracture cases. Bone.

[CR36] Wold S, Johansson E, Cocchi M (1993) PLS-partial least squares projections to latent structures in 3D-QSAR in drug design; theory methods and applications, pp 523–550

[CR37] Wold S, Trygg J, Berglund A, Antti H (2001). Some recent developments in PLS modeling. Chemom Intell Lab Syst.

